# Tularemia as a waterborne disease: a review

**DOI:** 10.1080/22221751.2019.1638734

**Published:** 2019-07-09

**Authors:** Aurélie Hennebique, Sandrine Boisset, Max Maurin

**Affiliations:** a Centre National de Référence des Francisella, Institut de Biologie et de Pathologie, Centre Hospitalier Universitaire Grenoble Alpes, Grenoble, France; b Université Grenoble Alpes, Centre National de la Recherche Scientifique, TIMC-IMAG, Grenoble, France

**Keywords:** Tularemia, *Francisella tularensis*, *Francisella* species, waterborne diseases, aquatic environment, mosquitoes, amoeba, bacterial biofilms

## Abstract

*Francisella tularensis* is a Gram-negative, intracellular bacterium causing the zoonosis tularemia. This highly infectious microorganism is considered a potential biological threat agent. Humans are usually infected through direct contact with the animal reservoir and tick bites. However, tularemia cases also occur after contact with a contaminated hydro-telluric environment. Water-borne tularemia outbreaks and sporadic cases have occurred worldwide in the last decades, with specific clinical and epidemiological traits. These infections represent a major public health and military challenge. Human contaminations have occurred through consumption or use of *F. tularensis*-contaminated water, and various aquatic activities such as swimming, canyoning and fishing. In addition, in Sweden and Finland, mosquitoes are primary vectors of tularemia due to infection of mosquito larvae in contaminated aquatic environments. The mechanisms of *F. tularensis* survival in water may include the formation of biofilms, interactions with free-living amoebae, and the transition to a ‘viable but nonculturable' state, but the relative contribution of these possible mechanisms remains unknown. Many new aquatic species of *Francisella* have been characterized in recent years. *F. tularensis* likely shares with these species an ability of long-term survival in the aquatic environment, which has to be considered in terms of tularemia surveillance and control.

## GRAPHICAL ABSTRACT

**Figure:**
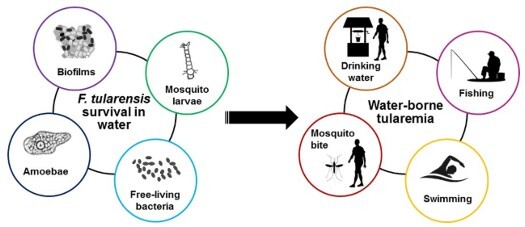

## Introduction

*Francisella tularensis* is a small, Gram-negative coccobacillus and the causative agent of the zoonosis tularemia [1]. Because it is highly infectious and can be spread by aerosols, this microorganism is classified in the category A of potential agents of biological threat by the US Centers for Disease Control and Prevention (CDC) [2]. The species *F. tularensis* is classically divided into four subspecies: *F. tularensis* subsp. *tularensis* (Type A strains), *F. tularensis* subsp. *holarctica* (Type B strains), *F. tularensis* subsp*. mediasiatica*, and *F. tularensis* subsp. *novicida*, which may be considered a different species (*F. novicida*) because it is an aquatic bacterium of low virulence in humans [3,4]. Type A and type B strains of *F. tularensis* are the two etiological agents of tularemia. The former subspecies is mainly localized in North America, although it has been occasionally detected in arthropods in Slovakia and Austria [5]. The latter one is found throughout the Northern Hemisphere [1], but has recently been detected in Australia [6].

*F. tularensis* has multiple reservoirs. Firstly, this bacterium can infect a multitude of animal species, including lagomorphs and small rodents, which are the primary sources of human infections [1,7,8]. Secondly, *Ixodidae* ticks are vectors but also a probable reservoir of *F. tularensis* owing to the transstadial transmission of this pathogen in these arthropods [1,8]. Mosquitoes and deer flies can also transmit *F. tularensis* to humans and animals in specific areas, but are not considered long-term reservoirs of this pathogen [1,8]. Finally, a hydro-telluric reservoir of *F. tularensis* is suggested by numerous studies but has not been extensively characterized [1,8]. Water-borne tularemia was first described in the 1930s in the USSR[9]. Human infection with *F. tularensis* may occur from these different reservoirs, and the clinical presentation of tularemia is primarily related to the mode of infection. Six major clinical forms of tularemia are classically recognized. The most frequent route of contamination is through the skin, through contact with an infected animal (especially hares in hunters) or arthropod bites. The ulceroglandular form of tularemia combines a skin ulcer at the site of *F. tularensis* inoculation and regional lymphadenopathy. The glandular form corresponds to regional lymphadenopathy without any visible skin lesion. Infection with *F. tularensis* through the conjunctiva (e.g. hand to eye contamination) or the oral route (ingestion of contaminated water or food) correspond to the oculoglandular and oropharyngeal forms, respectively. The involvement of lungs through inhalation of infected aerosols or hematogenous spread of bacteria corresponds to the pneumonic form. Finally, whatever the portal of entry of bacteria, severe sepsis often associated with confusion and *F. tularensis* bacteremia corresponds to the typhoidal form. The present review summarizes the literature data on human tularemia cases infected from aquatic sources, detection of *F. tularensis* in aquatic environments, and potential mechanisms of *F. tularensis* survival in water environments.

## Search strategy and selection criteria

Data on tularemia cases related to aquatic sources, on the presence of *Francisella* species in water environments, and the mechanisms of survival of these bacteria in water environments were collected from the English literature in the PubMed database. Only articles published in the last two decades (1998–2018) were included. They were extracted using the keywords ‘tularemia' or ‘*Francisella*' and ‘case report’ or ‘water' or ‘mosquito' or ‘biofilms' or ‘amoeba.' In addition, data on other aquatic *Francisella* species were added, including *F. novivida*, *F. philomiragia*, *F. halioticida*, *F. hispaniensis*, *F. noatunensis*, *F. salina, F. frigiditurris*, *Allofrancisella* (formerly *Francisella*) *gangzhouensis*, *F. marina*, *F. ulginis* and *F. endociliophora*.

## Tularemia of aquatic sources

### Tularemia and drinking water

In the last 20 years, tularemia cases linked to drinking water have been reported in Turkey, Kosovo, Bulgaria, Georgia, Macedonia, Norway, Sweden, Italy, and Germany (Table 1).

**Table 1.** Tularemia outbreaks and sporadic cases related to drinking water.

Country	Year of occurrence	Number of cases	Clinical forms (n)	Source of infection (water detection of *F. tularensis*)	*F. tularensis* subsp. involved	Reference
Bulgaria	1997–2005	285	OP (275), UG (6), OG (4)	Drinking water mainly from private wells (culture, pos)	*holarctica*	[40]
Georgia	2006	26	OP (21), GL (5)	Using water from the community water supply (culture, pos)	NS	[41]
Germany	2007	1	OP	Drinking surface water (NS)	*holarctica*	[49]
Italy	1988	24	OP (12), OG (1), AS (11)	Drinking water (NS)	NS	[48]
Kosovo	1999–2000	> 200	OP	Drinking water or eating food (Ag, neg)	NS	[39]
	2001–2002	> 200	OP	Drinking water or eating food (Ag, neg)	NS	[38]
Macedonia	2015	13	NS	Drinking water (NS)	NS	[42]
Norway	1997	8	OP or UG	Drinking water from wells (one well with a lemming carcass PCR-positive for *F. tularensis*) (PCR, pos)	NS	[43]
	2006	9	OP (5), GL (3), UN (1)	Drinking water from private wells or eating snow (PCR, pos)	NS	[44]
	2011	39	OP (21), UG or GL (10), PN (2), TY (2), AS (3), UN (1)	Drinking water from private wells or stream (PCR, pos)	NS	[45]
Sweden	NS	1	OP	Drinking water from a well (NS)	*holarctica*	[46]
	2013	6	OP	Drinking water from a private well (culture, pos)	NS	[47]
Turkey	1988–1998	205	OP (83%), OG (8%), UN (9%)	Drinking water (NS)	NS	[14]
	NS	1	OP	Drinking water from a well (culture, neg)	NS	[29]
	2001	14	OP	Drinking village pipe water (culture, neg)	NS	[35]
	2005	5	OP	Drinking natural spring water (PCR, neg)	NS	[30]
	2005	10	OP (7), AS (3)	Drinking spring water (culture, neg and PCR, pos)	NS	[20]
	2004–2005	54	OP	Drinking water (PCR, neg)	NS	[34]
	2004–2005	39	OP	Drinking water (NS)	NS	[36]
	NS	2	OP	Drinking water (NS)	NS	[28]
	2005	11	OP (8), OG (3)	Drinking village fountain water (PCR, pos)	NS	[19]
	2004–2005	145	OP	Drinking spring water (NS)	*holarctica*	[13]
	2005	70	OP mostly	Drinking natural spring water (culture, neg)	NS	[33]
	2004	86	OP mostly	Drinking water from a rivulet (PCR, pos)	NS	[17]
	2004–2005	135	OP	Drinking natural spring water (PCR, neg)	NS	[11]
	2000	22	OP (19), UG (3)	Drinking spring water (PCR positive for *Francisella* spp.)	NS	[18]
	2005–2006	58	OP	Drinking natural spring water (PCR, pos)	*holarctica*	[16]
	NS	1	OG	Drinking and washing face with spring water (NS)	NS	[26]
	NS	3	OP	Drinking spring water (NS)	*holarctica*	[27]
	NS	4	OP	Drinking water (NS)	NS	[23]
	2011	2	PN (with bacteremia)	Drinking water (culture, pos)	*holarctica*	[24]
	2010	4	OP (3), OP and OG (1)	Drinking (3) or exposure (1) to natural spring water (NS)	NS	[25]
	2009–2011	139	OP (74%), GL (15.8%), OG (5%)	Drinking spring water (PCR, pos)	NS	[15]
	NS	2	OP (with abdominal lymphadenopathy)	Drinking natural spring water (NS)	NS	[21]
	NS	3	OP	Drinking natural spring water (NS)	NS	[22]
	2010–2012	110	OP or GL	Drinking water (PCR, pos)	NS	[12]
	2013	55	OP	Drinking tap water contaminated by surface water (culture, neg)	NS	[31]
	2008, 2009 and 2012	89, 54 and 35	OP	Drinking water (culture, pos)	NS	[32]

(NS) Not specified; Clinical forms: ulceroglandular (UG), glandular (GL), oropharyngeal (OP), oculoglandular (OG), pneumonic (PN), typhoidal (TY), asymptomatic (AS), and unknown form (UN); (Ag) *F. tularensis* antigen detection; (pos) positive, (neg) negative.

In Turkey, tularemia reemerged in 1988 in the form of water-borne outbreaks of oropharyngeal tularemia cases [10]. Between 1988 and 2018, 28 tularemia outbreaks and non-outbreak tularemia cases linked to consumption of contaminated water were reported in this country [11–36]. The Turkish outbreaks were frequently of large scale, involving more than one hundred people for some of them [11–15]. Patients were almost exclusively suffering from the oropharyngeal form of tularemia, in accordance with the oral route of contamination with *F. tularensis*. In addition, cases were observed in the whole population, with a male/female sex ratio close to 1 or even lower, and both children and adults involved [11–19,31,33–35]. This is in sharp contrast to countries where infections usually occur through contact with animals or tick bites, and therefore predominate in middle-aged men due to more frequent occupational or leisure exposures [8]. Less frequently, exposure to contaminated water resulted in glandular [12,15], oculoglandular [14,15,19,25,26], and pneumonic [24] forms of tularemia. In some reports, *Francisella* species or more specifically *F. tularensis* were detected by PCR or culture in the suspected water sources [12,15–20,24,32]. Moreover, the same *F. tularensis* genotypes were detected concomitantly from water sources and related tularemia patients in some studies [24,32,37], confirming the epidemiological link between drinking water and human infection. In the majority of cases, the source of contamination was spring water, water from the community water supplies or water from wells. These water sources were not or inadequately disinfected. Contamination of the water by infected animal carcasses or excreta was the most likely hypothesis [10]. Although *F. tularensis* subsp*. holarctica* was identified only for some of these outbreaks, only type B strains have been associated with human infections in Europe and Asia.

In Kosovo, two huge oropharyngeal tularemia outbreaks occurred in 1999–2000 and 2001–2002, each one involving more than two hundred patients [38,39]. It was not possible to establish if the primary source of infection was contaminated water or food, but it can be assumed that both were involved in the human transmission of *F. tularensis* [38,39]. These outbreaks were likely a collateral damage of the Kosovo war, which ended in 1999 and left the country with bad sanitary conditions. Indeed, people reported that mice and rats invaded their villages during this period, which may correlate with contamination of water wells and cellars with *F. tularensis*. This bacterium was detected in a field mouse recovered from a water well in an affected village [39].

A long-lasting outbreak occurred between 1997 and 2005 in Bulgaria, involving 285 people [40]. *F. tularensis* was isolated from water samples collected from private wells. Thus, the route of human contamination was likely water consumption. The majority of patients suffered from the oropharyngeal form of tularemia [40]. Similarly, in Georgia, a tularemia outbreak involving 26 patients occurred in 2006 [41], with a predominance of oropharyngeal cases. The water source was the community water supply from which *F. tularensis* was isolated [41]. Finally, a small outbreak involving 13 patients was described in 2015 in Macedonia [42].

Water-borne tularemia cases are also reported in Scandinavia. In Norway, three small outbreaks, involving eight to 39 people, linked to the consumption of contaminated water were reported over 20 years [43–45]. Most of the patients suffered from oropharyngeal tularemia [43–45], although ulceroglandular, glandular, pneumonic and typhoidal forms were also observed [43–45]. The source of these infections was water collected from private water wells, as confirmed by PCR-detection of *F. tularensis* DNA [43–45]. *F. tularensis*-infected rodent carcasses probably contaminated these water wells. In one report, a lemming carcass retrieved from the contaminated water well was PCR-positive for *F. tularensis* [43]. The involved *F. tularensis* subspecies was not identified, but only type B strains are found in this country*.* In Sweden, a small outbreak and sporadic cases of oropharyngeal tularemia were also associated with the consumption of contaminated water from private water wells [46,47].

Tularemia cases related to *F. tularensis*-contaminated water consumption have been occasionally observed in other countries. In Italy, in 1988, a tularemia outbreak linked to the use of water collected from an aqueduct involved 24 people living in the same village, most of which developed oropharyngeal tularemia [48]. More recently, a German oropharyngeal case of tularemia was suspected to be associated with drinking of non-purified surface water during a holiday in Bavaria [49].

### Tularemia and mosquitoes

In Sweden and Finland, tularemia is mainly transmitted through mosquito bites. Large tularemia outbreaks, frequently involving more than one hundred people, have occurred almost annually in Sweden during the last two decades [46,50–54]. These outbreaks mainly occurred in central Sweden (counties of Örebro, Stockholm, Södermanland, Västmanland, Värmland, and Dalarna), in wet and woody areas of the Boreal Forest, during the late summer period, because of the mosquito vector habitat and seasonality [46,50–54]. A large scale epidemiological study in Sweden over 29 years identified that tularemia incidence was positively correlated with the presence of lakes and rivers [55]. Similarly, outbreak modeling demonstrated that tularemia in Sweden is concentrated in a few high-risk regions, with a high incidence in summer likely related to the population dynamics of the mosquito vector [56]. Due to this specific mode of transmission, the ulceroglandular form of tularemia is predominant in Sweden. In addition, cases occur in the exposed population regardless of age, but more frequently in men than women probably because of more outdoor activities in men [46,50,51,53]. Swedish mosquitoes collected in Örebro were PCR-positive for *F. tularensis* subsp. *holarctica*, still arguing their role in tularemia transmission in this country [57].

Tularemia is also predominantly a mosquito-borne disease in Finland, where several tularemia outbreaks [58] or sporadic cases [59,60] have been linked with this mode of transmission. Here again, tularemia is a seasonal disease occurring during late summer, and ulceroglandular tularemia is the primary clinical presentation [58,61].

The potential role of mosquitoes in the transmission of *F. tularensis* to humans has been occasionally reported in other countries, such as Germany [62]. All these reports involved *F. tularensis* subsp. *holarctica* as they occurred in Europe. Table 2 summarizes the literature reports of mosquito-borne tularemia cases. Interestingly, *F. tularensis* DNA was detected in Alaskan mosquitoes, suggesting that tularemia could be a mosquito-borne disease in this U.S. state [63].

**Table 2.** Tularemia outbreaks and sporadic cases related to mosquito bites.

Country	Year of occurrence	Number of cases	Clinical forms	*F. tularensis* subsp. involved	Reference
Finland	2000	1	UG	*NS*	[59]
	2007	50	UG mostly	*holarctica*	[58]
	2010	1	UG	*NS*	[60]
Germany	NS	1	UG	*holarctica*	[62]
Sweden	1981, 1995, 1999, 2000, 2002, 2003, and 2004	349	UG mostly	*holarctica*	[50]
	2000–2004	278	UG	*holarctica*	[46,51]
	2000	105	UG mostly	*NS*	[52]
	2003	475	NS	*NS*	[53]
	2006	90	NS	*NS*	[54]

(NS) Not specified; Clinical forms: ulceroglandular (UG).

### Tularemia of other aquatic sources

All around the world, human contamination with *F. tularensis* has occasionally occurred through other types of water exposure (Table 3).

**Table 3.** Tularemia outbreaks and sporadic cases related to other aquatic sources.

Country	Year of occurrence	Number of cases	Clinical forms (n)	Source of infection	*F. tularensis* subsp. involved	Reference
Canada	NS	1	UG	Suspicion of finger injury while cleaning freshwater fishes	*NS*	[70]
Finland	NS	1	PN	Near-drowning accident	NS	[65]
France	NS	1	PN with bacteremia	Near-drowning accident while fishing in a river	*holarctica*	[64]
	2008–2014	3	Otomastoiditis	Canyoneering in the same river	*holarctica*	[68]
Spain	1998	19	UG and GL	Crayfish fishing in a river	*holarctica*	[69]
Turkey	2007	3	OP	Swimming in the same lake	*NS*	[67]
	2010	1	GL	Swimming in freshwater	NS	[66]
USA	2000–2006 (Martha’s Vineyard island)	59	PN (38), UG or GL (9), TY (2), OP (1)	Water environmental source likely	*tularensis*	[73,74]
	2016	1	UG	Finger injury while fishing in a freshwater lake.	*holarctica*	[71]

(NS) Not specified; Clinical forms: ulceroglandular (UG), glandular (GL), oropharyngeal (OP), pneumonic (PN), and typhoidal (TY).

Tularemia cases were reported after near-drowning accidents in France [64] and Finland [65]. For these two cases, patients developed a pneumonic form of the disease after inhalation of contaminated water. For the French case, contamination occurred after inhalation of freshwater and was caused by *F. tularensis* subsp. *holarctica.* The involved subspecies and water salinity were not specified for the Finnish case. Four tularemia cases reported in Turkey were related to swimming activities [66,67]. Three of these cases were oropharyngeal forms that developed after swimming in the same lake [67]. The fourth patient suffered from a glandular form after swimming in a natural aquatic environment in a valley [66]. Otomastoiditis caused by *F. tularensis* subsp. *holarctica* were reported in three patients (two from France, one from Germany) after canyoning in the same river in France, between 2009 and 2014 [68]. Otitis likely occurred after exposure to *F. tularensis* contaminated water, although the source was not specifically identified [68].

Tularemia cases also occurred after handling *F. tularensis*-contaminated aquatic animals [69–71]. A small tularemia outbreak involving 19 patients occurred in Spain after crayfish fishing [69]. The patients developed a glandular or ulceroglandular form of the disease through skin injuries while catching or cleaning red swamp crayfishes sinned in the same river. Cases were grouped over three weeks after which fishing was prohibited [69]. The source of contamination was confirmed by PCR-detection of *F. tularensis* DNA in one crayfish and two water samples from the river [69]. In Canada, a patient developed ulceroglandular tularemia after a finger injury while cleaning pike and pickerel, which are freshwater fishes [70]. Similarly, in the USA, a patient contracted an ulceroglandular tularemia due to *F. tularensis* subsp. *holarctica* after a finger injury while fishing on a freshwater lake [71].

The particular ecology of Martha’s Vineyard (Massachusetts), an island in the east coast of the USA should be highlighted. Two outbreaks of pneumonic tularemia occurred on this island, the first in 1978 [72] and the second from 2000 to 2006 [73,74]. *F. tularensis* subsp. *tularensis* was isolated from one dead patient and rabbits [73,75]. According to a case–control study, landscaping activities, especially lawn mowing and brush-cutting, were the most likely sources of exposure to *F. tularensis* through aerosols from environmental material [73,76]. However, the environmental reservoir of *F. tularensis* could not be characterized. Skunks and raccoons were found to be seropositive for *F. tularensis*, but other animal species tested had not or very rarely been infected with this pathogen [75]. A low prevalence of infection and a high degree of *Francisella* genetic diversity in ticks argued against the role of these arthropods as a source of human contamination [77]. In contrast, the role of the aquatic environment as a long-term reservoir of *F. tularensis* was strongly suspected. PCR detection of *Francisella* sp. DNA was frequently reported from brackish-water samples collected on the island [78].

The role of brackish water as a potential reservoir of *F. tularensis* is supported by a more recent study in Nunavik territory (Canada) demonstrating that tularemia seroprevalence in the human population was positively correlated with residence near the coast [79].

## Other *Francisella* species and aquatic reservoir

The genus *Francisella* includes many other species with a proven or probable aquatic habitat. Genetic analyses have suggested that the ancestral *Francisella* species originated in a marine habitat [80]. Here, we review water-borne human infections caused by *Francisella* species other than *F. tularensis* (Table 4). Some of these species are opportunistic pathogens mainly causing diseases in immunocompromised patients exposed to an aquatic environment. Among them, infections caused by *F. philomiragia* or *F. novicida* have been most frequently reported.

**Table 4.** Human infections caused by *Francisella* species other than *F. tularensis* related to aquatic sources.

Country	Year of occurrence	Number of cases	Clinical forms	Source of infection	*F. tularensis* subsp. involved	Reference
Australia	NS	1	UG	Cut in the toe in brackish water	*F. novicida-like*	[99]
	NS	1	NS, bacteremia	Cut with a fishhook while fishing	*F. hispaniensis*	[105]
Canada	NS	1	OP and PN	Skin abrasion from a saltwater crab	*F. philomiragia*	[84]
Spain	NS	1	OP	Holiday activities in the Mediterranean sea	*F. philomiragia*	[83]
Turkey	NS	1	TY	Swimming in the sea and taking mud baths	*F. philomiragia*	[81]
USA	1977–1985	5	PN, bacteremia	Near-drowning accident in saltwater or brackish water	*F. philomiragia*	[86]
	1995	1	NS, bacteremia	Use of water from a private well for food preparation and bathing	*F. novicida-*like	[100]
	NS	1	NS, bacteremia	Working in the brackish water of Assawoman Bay in Maryland	*F. philomiragia*	[85]
	NS	1	TY	Practice of jet-ski in a bay in the Atlantic Ocean	*F. philomiragia*	[82]
	NS	1	NS, bacteremia	Near-drowning accident while surfing in the Atlantic Ocean	*F. novicida*	[97]
	2001	1	NS	Exposure to hot spring water near a salt-lake	*F. novicida*	[89]
	NS	1	UG	Suspicion of infection through contact of an open wound in brackish water	Species close to *F. halioticida*	[113]
	2011	3	NS, bacteremia	Consumption of ice from ice machines	*F. novicida*	[98]

(NS) Not specified; Clinical forms: ulceroglandular (UG), glandular (GL), oropharyngeal (OP), oculoglandular (OG), pneumonic (PN), and typhoidal (TY).

Less than 20 human infections with *F. philomiragia* have been published in the English literature [81–88]. These infections occurred in healthy individuals that have survived a near-drowning accident [86] or in immunocompromised patients (especially people suffering from chronic granulomatous disease) after exposure to an aquatic environment [81–86]. Interestingly, human contamination usually occurred after exposure to salt- or brackish-water [81–86]. Infections with *F. philomiragia* most often occurred during recreational activities in sea, ocean or bay connected to the ocean [81–83,85,86]. A young patient was contaminated after a skin lesion caused by a saltwater crab [84]. In a review of 14 cases, Wenger *et al.* showed that most *F. philomiragia* infections occurred in patients living within 50 miles of a salt-water coastline [86], again suggesting a clear association between salt-water exposure and *F. philomiragia* infections.

The geographical distribution of *F. philomiragia* is probably wide as human infections with this species have been described in the USA [82,85,86], Canada [84], Europe [83] and Turkey [81]. Frequent detection of *F. philomiragia* in water samples by culture or PCR indicates that the aquatic environment is likely the primary reservoir of this bacterium [78,89–93]. In two studies, in Norway [92] and the USA [78], *F. philomiragia* was only found in salt- or brackish-water but not in fresh-water, suggesting that water salinity is a major element in the natural life cycle of *F. philomiragia*. However, this species was also isolated from spring water near a salt lake in the USA [89] and cooling towers in China [91], reflecting its distribution in different aquatic reservoirs. *F. philomiragia* DNA was also detected in ballast water from cargo traveling all around the world [90], which might be a mode of diffusion of this species at the global scale. Although the aquatic reservoir appears to be predominant, *F. philomiragia* was isolated in a sick muskrat and in brackish water surrounding it [94] suggesting that a mammal reservoir may exist for this bacterium. *F. philomiragia* was also detected in *Dermacentor* ticks [95].

*F. novicida* is also a rare human pathogen. A dozen cases have been published in the English literature so far [96]. For all cases with an identified mode of contamination, an aquatic source was involved [89,97–100]. In the USA, a case of *F. novicida* bacteremia occurred after a near-drowning accident in the Atlantic Ocean [97], another case after exposure to hot spring water near a salt lake [89], and the last one after exposure to water from a private water well [100]. A striking outbreak of *F. novicida* bacteremia occurred among inmates in the USA after consumption of ice from ice machines from which *F. novicida* DNA was detected by PCR [98]. Finally, in Australia, a patient developed an ulceroglandular form of infection with *F. novicida* after cutting himself in brackish water [99]. Most of the patients suffering from *F. novicida* infection were immunocompromised or had underlying health conditions [98,100]. The identification of *F. novicida* has never been reported in animals or arthropods [96]. The only known reservoir of this bacterium is the aquatic environment as attested by its repeated isolation from water samples [78,89,101,102]. As for *F. philomiragia*, salinity seems to impact *F. novicida* survival in water, as this bacterium was detected by culture or PCR only in sea-water [101,102], brackish-water [78] and spring water near a salt lake [89].

Other *Francisella* species have been rarely associated with human infections originating from aquatic sources. *F. hispaniensis* was first isolated in 2003 from a Spanish patient suffering from bacteremia [103,104]. The source of contamination was not identified [103]. However, a few years later, *F. hispaniensis* was isolated in the blood of an Australian immunocompromised patient after he cut himself with a fishhook while fishing [105].

The genus *Francisella* also includes species that are pathogenic for marine animals. *F. noatunensis* subsp. *orientalis* and *Francisella noatunensis* subsp. *noatunensis* are widely described as warm- and cold-water fish pathogens, responsible for ‘piscine francisellosis.’ This disease causes high morbidity and mortality in many fish species worldwide and is responsible for economic losses in aquaculture [106–109]. *F. noatunensis* does not seem to be pathogenic in humans [106]. *F. halioticida* can infect *Haliotis* mollusks (abalones) [110,111] and *F. marina* *sp. nov.* was recently identified as causing disease in Spotted Rose Snapper fishes [112]. Interestingly, a novel *Francisella* species very close to *F. halioticida* was isolated in the USA, in a diabetic patient, from an infected skin wound developed after contact with brackish water [113]*.* Finally, other *Francisella* species are endosymbionts of marine ciliates such as *F. endociliophora* [114].

Over the past ten years, new *Francisella* species have been isolated from the aquatic environment. *F. salina* and *F. uliginis* were isolated from sea-water in the USA [101,102]. Water from cooling towers also seems to be a reservoir of *Francisella* species as attested by recent isolation from these air conditioning systems of *F. frigiditurris* in the USA [102] and *F. guangzhouensis* in China and Germany [91,115–117]. These two latter species have been transferred to the new genus *Allofrancisella* [115].

Table 5 summaries studies dealing with the detection of *Francisella* species in water samples, either using culture, PCR or both methods. It shows the broad spectrum of *Francisella* species found in aquatic reservoirs. Regarding PCR methods, it is important to notice that *Francisella* species that are not yet characterized may not be PCR-amplified from water samples due to the use of inadequate primers [78]. Even if there is PCR amplification with a new species, it may not be accurately identified as a novel species due to a lack of resolution within the utilized amplicon. Full identification of a novel species requires whole genome sequencing.

**Table 5.** Detection of *Francisella* species in water samples.

Country	Year of sampling	Type of water samples (n)	Testing methods	Findings (n samples)	Reference
China	2008	Cooling towers (NS)	Culture and strain identification by fatty acid analysis, and 16S rRNA, 23S rRNAs, *recA, rpoA*, *rpoB*, *rpoD*, *rpoH*, *groEL*, *dnaK*, *gyrB*, *sdhA*, and *fopA* genes sequencing	*F. guangzhouensis* (4)	[117]
	2009–2011	Cooling towers (312)	Culture and strain identification by 16S rRNA, *rpoB* and *sdhA* genes sequencing	*Francisella* strains phylogenetically close to *F.* philomiragia (1) or *F. guangzhouensis* *(8)*	[91]
	2008 and after	Cooling towers (NS)	Culture and strain identification by mass spectrometry, fatty acid analysis, and 16S rRNA, *rpoB, mdhA,* and *sdhA* genes sequencing	*Francisella* strains phylogenetically close to *F.* guangzhouensis (5). Description of *Allofrancisella inopinata* gen. nov., sp. nov. and *A. frigidaquae* sp. nov.; transfer of *F. guangzhouensis* to A. *guangzhouensis* comb. nov.	[115]
Germany	2005–2006	NS (28)	PCR targeting 16S rRNA and *fopA* genes	*F. tularensis* (1)	[132]
	2012	Cooling tower (NS)	Culture and strain identification by 16S rRNA, *fopA, gyrA, rpoA, groEL, sdhA,* and *dnaK* genes sequencing	*Francisella* strain phylogenetically close to *F. guangzhouensis* (*1)*	[116]
Netherlands	2013–2017	Surface water collected from areas with reported human or hare tularemia cases (127) or unrelated to recent tularemia cases (339)	PCR targeting *ISFtu2* and *fopA* genes	*F. tularensis* in 88% of the case-related samples and in 10% of the randomly collected samples	[128]
Norway	2010	Seawater (149) or freshwater (64)	PCR sequencing of 16S rRNA; and for positive samples *sdhA* and *purCD* PCR	*Francisella* sp. in seawater (38) but not in freshwater samples. *F. philomiragia*-related species mostly.	[92]
Sweden	2003– 2005	Surface water (341)	PCR sequencing of *lpnA,* 16S rDNA, *lpnA,* and FtM19InDel	*F. tularensis* (108), mainly subsp. *holarctica*, rarely subsp. *mediasiatica*	[131]
Turkey	2008 - 2009	Rivers, spring waters or village fountains in tularemia-endemic areas (154)	Culture and strains identification by 16S rRNA gene sequencing; and PCR targeting IS*Ftu2*	*F. tularensis* subsp. *holarctica* isolation (4); or *F. tularensis* positive PCR IS*Ftu2* (17)	[130]
Ukraine	1941– 2008	NS (NS)	Culture	*F. tularensis* (393)	[129]
USA	2003	NS (23)	PCR sequencing of 16S rDNA; and for positive samples *ISFtu2*, *23 kDa, tul4, fopA* and *sdhA* PCR	*F. philomiragia* (1)	[93]
	NS	Seawater (NS)	Culture (CHAB-PACCV medium) and strains identification by PCR sequencing of 16S rRNA and *sdhA* genes	*F. philomiragia*-like (2) and *F. novicida*-like (1). The two *F. philomiragia*-like strains were latter characterized as new *Francisella* species by Challacombe *et al.*: *F. salina* and *F. uliginis.*	[101,102]
	2005– 2007 (Martha’s Vineyard island)	Fresh-water (35) or brackish-water (42)	Culture and PCR targeting 16S rRNA gene; for positive samples *sdhA, tul4*, IS*Ftu2*, and *fopA* PCR.	No positive fresh-water samples. *Francisella* DNA detected by PCR in brackish-water samples (19). Mainly *F. philomiragia*; few *F. novicida*- or *F. tularensis*-like strains. *F. philomiragia* grown from one brackish-water sample.	[78]
	NS	Hot or cold spring waters near a salt lake (NS)	Culture and strain identification by fatty acid analyses, ribotyping and 16S rRNA gene sequencing	*F. philomiragia* and *F. novicida* (NS)	[89]
	NS	Cooling tower (NS)	NS	*F. frigiditurris* (NS)	[102]
Cargo ships*	2007–2008	Ballast water from 5 general cargo ships (NS)	PCR sequencing of 16S rRNA gene	*F. philomiragia* and *F. noatunensis* in ballast water from 4 cargo ships	[90]

(NS) Not specified; * Cargo ships from Columbia, Republic of the Congo, USA, Canada, and Iran.

## Mechanisms of *F. tularensis* survival in water environments

The high frequency of water-borne tularemia cases implies the persistence of *F. tularensis* in the aquatic environment. However, the mechanisms of *F. tularensis* survival in this environment have not been elucidated so far.

### Long-term survival of F. tularensis in water

Experimental studies suggest long-term survival of *F. tularensis* in various water environments. Several authors described the survival of *F. tularensis* in water microcosm from 1 to 70 days [118–120]. *F. tularensis* survival seems to be influenced by both water temperature [118] and salinity [119]. Gilbert and Rose observed that *F. tularensis* subsp. *holarctica* remains cultivable after a stay in water for one day at 5°C or 25°C, but up to 28 days at 8°C [118]. Berrada and Telford showed that both type A and type B strains of *F. tularensis* remain cultivable after a stay of 8–10 days in fresh-water, but 30–42 days in brackish-water, both at 21°C [119]. In another study, *F. tularensis* subsp. *holarctica* remained cultivable after a stay in fresh water at 8°C up to 70 days [120].

Interestingly, when *F. tularensis* became uncultivable on agar plates, bacteria could still be detected and were metabolically active in water [118,120]. This phenomenon was observed by Gilbert and Rose [118] and Forsman *et al.* [120] for *F. tularensis* subsp. *holarctica.* The latter authors described the persistence of metabolic activity for *F. tularensis* in water up to 140 days [120]. This state is defined as ‘viable but non-culturable' (VBNC) and could be responsible for long-term survival of bacteria in the water environment. The VBNC state has been defined as a state from which bacterial cells cannot be cultured but maintain a metabolic activity and cellular integrity [118]. In addition, the VBNC state may be reversible, as bacteria may become cultivable under certain conditions. This reversion in the ability to grow on acellular media is called ‘resuscitation' of VBNC bacteria. The VBNC state has been described for a wide range of bacteria such as *Vibrio* sp. [121,122], *Campylobacter* sp. [123], *Escherichia coli* [122] and *Legionella pneumophila* [124]. Depending on the bacterial species, the infectious nature and pathogenic potential of VBNC cells are variable, as well as the ways of their resuscitation. Forsman *et al.* reported that *F. tularensis* VBNC cells were no longer virulent in mice, and could not be resuscitated [120]. Finally, a recent study demonstrated that *F. tularensis* subsp. *holarctica* possesses a mechanosensitive channel that protects this bacterium from hypo-osmotic shock when it is released from an infected animal to water [125].

Long-term survival in water has also been reported for other *Francisella* species. Berrada and Telford showed that *F. novicida* (like *F. tularensis*) remains cultivable after a stay of up to 30–42 days in brackish-water, at 21°C [119]. The fish-pathogen *Francisella* species are able to survive in water in the absence of a suitable fish host. Indeed, *F. noatunensis* subsp. *orientalis* remains cultivable after a stay up to 2 days in freshwater and up to 3 days in seawater [126]. *F. noatunensis* subsp. *noatunensis* remains cultivable after up to 12 days in freshwater and up to 50 days in seawater [127]. Interestingly, *F. noatunensis* was also able to enter in a VBNC state after a period of stay in the water, and these VBNC were not pathogenic to cods [127].

Long-term survival of *F. tularensis* in natural aquatic environments is suggested by a number of studies detecting this bacterium by culture or PCR in environmental water samples [12,16,17,19,20,32,40,41,43,44,47,78,128–132]. Not surprisingly, long-term detection of *F. tularensis* in water environments was reported in countries were water-borne tularemia cases are frequent and predominant. In Turkey, two environmental studies identified *F. tularensis* subsp. *holarctica* in water samples collected from the aquatic environment or from village water supply systems highlighting the role of different water sources as common and persistent reservoirs of *F. tularensis* in this country [32,130]. In Sweden, *F. tularensis* subsp. *holarctica* was PCR-detected in water samples collected during outbreak and non-outbreak periods, in tularemia endemic areas [131]. However, *F. tularensis* was also detected from water samples in countries where tularemia is not or rarely a water-borne disease [128,129,132]. Hightower *et al.* isolated *F. tularensis* strains from water in Ukraine and considered that the aquatic environment was the third main *F. tularensis* reservoir after arthropods and mammals in this country [129]. In Germany, following the reemergence of tularemia in 2004, an ecological study in outbreak areas found a river water sample PCR-positive for *F. tularensis* suggesting a natural aquatic reservoir for this bacterium [132]. In the Netherland, tularemia also re-emerged in 2011 leading to the surveillance of *F. tularensis* prevalence in the environment [128]. Surface water samples were PCR-positive for *F. tularensis* subsp. *holarctica* in 10% of the randomly collected samples and 88% of the samples collected in areas where tularemia cases among hares or humans had been reported [128]. These studies show the almost constant presence of *F. tularensis* in randomly collected water samples at different time periods. This observation can only be explained by repeated contaminations of the aquatic environment, particularly from the animal reservoir during epizootics, but also by the persistence of these bacteria in the water environment during non-epizootic periods.

Table 5 summarizes studies dealing with *F. tularensis* detection in water environments. PCR was more effective than culture for detection of *F. tularensis* in water samples, possibly because of a low bacterial inoculum, the encroachment of *F. tularensis* by other bacterial species, or a VBNC state of bacteria. However, PCR results should be interpreted with caution since it has been shown that *F. tularensis* specific primers (such as those targeting *fopA* or *tul4* genes) may also amplify DNA from *Francisella* species other than the tularemia agents [78].

### Survival in biofilms

An essential mechanism for survival and persistence of bacteria in the water environment is bioﬁlm formation. Biofilms are defined as naturally formed adherent communities of bacteria within an extracellular polymeric matrix [133]. A number of bacterial species, such as *Vibrio cholerae* [134], *Legionella pneumophila* [135], *Helicobacter pylori* [136] *and Pseudomonas aeruginosa* [137] form biofilms to promote their survival under environmental water conditions. *In vitro* studies have demonstrated that both *F. tularensis* subsp. *holarctica* [138,139] and *F. tularensis* subsp. *tularensis* [139] can form biofilms. The aquatic *Francisella* species, *F. novicida* [139–141] and *F. philomiragia* [142] have also been capable of biofilm formation experimentally. *F. novicida* was also demonstrated to be able to form biofilm in chitin surface, the second most abundant biopolymer in nature, providing the structure of arthropods, insects, and fungi [139]. *F. philomiragia* was shown to form more biofilm at 25°C than at 37°C, which is compatible with its natural aquatic reservoir [142]. The fish pathogen *F. noatunensis* was also demonstrated to form biofilm *in vivo.* [143]. Until now, biofilm formation has not been associated with virulence in *Francisella* species [133]. Thus, biofilm formation is most likely a way of environmental survival and persistence in these species [133]. To our knowledge, *Francisella* biofilm has never been described in natural environmental water microcosm.

### Survival in amoebae

Free-living amoebae are ubiquitous organisms in soil and water environments. Several human pathogens, such as *L. pneumophila* and some *Mycobacterium* species resist phagocytosis and digestion by the free-living amoebae and may survive in water environment inside amoebae [144]. Moreover, these bacteria may survive for long periods in amoeba cysts [144]. This mode of survival may apply to *Francisella* species, especially *F. tularensis*. Multiple experimental studies have focused on the interaction between *Francisella* species and several amoeba species [145–154]. Berdal *et al.* demonstrated that *F. tularensis* could penetrate in the amoeba *Acanthamoeba castellanii* and be released from it [152]. Then, other authors described the multiplication of *F. tularensis* subsp. *tularensis* in *A. castellannii* 24 h post-infection [150]. *F. novicida* and *F. philomiragia* were also able to multiply within *Hartmanella vermiformis* and *A. castellanii* [142,146,148,150,151]. *F. noatunensis* was also able to infect and replicate within the amoeba *Dictostelium discoideum* [149]. *Francisella* cells were localized within vacuoles in amoeba trophozoites [146] but were also able to survive in amoebal cysts for several weeks [150]. This latter finding suggested that amoeba could be a long-term reservoir of *Francisella* spp. in water environments. Other authors described an enhanced survival of *F. tularensis* subsp. *tularensis*, *F. tularensis* subsp. *holarctica* and *F. novicida* in co-culture with amoebae such as *A. castellanii*, *A. polyphaga*, *Vermamoeba vermiformis* or *Ochromonas danica* [145,147,153]. These authors described the presence of *Francisella* sp. inside and outside the amoeba in the co-culture model, suggesting that bacterial survival could be related to intra-amoebal replication, to favorable interaction between extracellular bacteria and amoeba, or both [145,147,153]. Interestingly, Gustafsson *et al.* demonstrated that growth supernatant of *A. palestinensis* (without the presence of amoebae) increased multiplication of *F. tularensis* [154]*.* After five days in co-culture with amoeba, *F. tularensis* subsp. *holarctica* was also shown to enter in a VBNC state [147]. In contrast to *L. pneumophila,* passage through amoebae did not increase *Francisella novicida* virulence [148]. Despite some disagreements about interaction mechanisms, all these reports argue that *Francisella* spp. are resistant to free-living amoeba and that protists may contribute to the survival of *Francisella* sp. in the water environment. However, to our knowledge, *Francisella* species have never been detected within amoebae in environmental water samples, in contrast to *L. pneumophila* and *Mycobacterium* spp. [155].

### Survival in mosquito larvae

Mosquito larvae may also represent a long-term *F. tularensis* reservoir in the aquatic environment. It has been shown that these larvae can ingest *F. tularensis* subsp. *holarctica* that are present in water and ingested bacteria survive throughout the different maturation stages of these arthropods up to adult mosquitoes [57,63,138,156,157]. The fact that mosquito-borne tularemia cases have occurred over years in Sweden and Finland should be considered a further evidence of the existence of a long-term aquatic reservoir of *F. tularensis.* However, it should be notified that mosquitoes could also be infected at the adult stage after a blood meal on an infected host [57]; in this case tularaemia is not water-borne.

## Discussion

Although water-borne tularemia was first described in the 1930s [9], this route of human contamination has been largely underestimated. Tularemia cases linked to the aquatic reservoir are common and can occur as large epidemics. Thus, tularemia is a major public health problem in countries where water-borne tularemia cases predominate. These cases may occur through consumption of *F. tularensis*-contaminated drinking water, such as in Turkey [11–36] and its neighboring countries [38–42], and in Norway [43–45] where large-scale tularemia outbreaks caused by *F. tularensis* subsp. *holarctica* are regularly reported. Drinking water as a source of human infections with type B strains of *F. tularensis* have also been occasionally encountered in Sweden [46,47] and central Europe [48,49]. Human infections have occurred after consumption of contaminated water from the community water supplies, especially in countries with bad sanitary conditions, old water networks and inappropriate water treatment. Consumption of unsanitized surface water or well water was also a source of contamination. Consequently, it is of primary interest for practitioners to keep in mind that tularemia may correspond to sub-acute or chronic pharyngitis associated with cervical lymphadenopathy, especially in patients living or traveling in countries where water-borne tularemia cases are frequent. In addition, medical questioning regarding tularemia exposure should include a statement about unsanitized water consumption. Until now, this mode of human contamination has been observed in restricted areas and only linked to *F. tularensis* subsp. *holarctica*. However, similar cases could occur throughout the Northern hemisphere where type B strains are encountered. *F. tularensis* subsp. *tularensis* has also been associated with the water environment [72–74], and could also cause tularemia cases related to drinking water.

Mosquito-borne tularemia is also related to contamination of the aquatic environment by *F. tularensis*. Tularemia outbreaks caused by *F. tularensis* subsp. *holarctica* in Sweden [46,50–54] and Finland [58–60] are primarily related to mosquito bites. Because of skin inoculation of bacteria, the ulceroglandular form of tularemia predominates. Infections usually occur during the warm season at the time of maximum activity of mosquitoes. Scandinavian practitioners are now particularly aware of this situation and usually diagnose tularemia early in the course of the disease. In contrast, this mode of transmission is most often unknown by physicians in other parts of the word, and mosquito-borne tularemia cases could be missed in patients returning from Scandinavian countries. Also, potential transmission of tularemia through mosquito bites outside Scandinavia has not been thoroughly evaluated. A probable autochtonous mosquito-borne tularemia case was reported in Germany [62]. In Alaska, mosquitoes tested positive for *F. tularensis* DNA [63] arguing that these arthropods could also transmit tularemia to humans in this US state. In this time of global warming, mosquito vectors could spread to new geographic areas, leading to a rise in mosquito-borne tularemia cases [158].

Finally, tularemia cases may occur through other types of aquatic exposure such as near drowning accident [64,65], swimming [66,67], canyoning [68] and fishing activities [69–71], due to penetration of *F. tularensis* through the skin, conjunctiva, or digestive and respiratory tracts. These cases have been reported in Europe [64,65,68,69], Turkey [66,67] and North America [70,71], suggesting a wide aquatic distribution of *F. tularensis*. Type B strains of *F. tularensis* were likely involved in all these cases, except one case occurring in Canada for which type A and type B strains could be involved [70]. These data suggest that *F. tularensis* subsp. *holarctica* could be more frequently associated with aquatic reservoirs than *F. tularensis* subsp. *tularensis* There is currently no explanation regarding the preferential association of type B strains with water. Williamson *et al.* demonstrated that type B strains can resist to hypoosmotic shock when released into water [125]. It would be interesting to compare the osmotic shock resistance of type A versus type B strains. Noticeably, almost all human contaminations occurred after contact with fresh-water, suggesting that *F. tularensis* may better survive in such aquatic environment. *F. tularensis* subsp. *tularensis* may also infect humans through water exposure, as attested by the particular ecology of the Martha’s Vineyard island in the USA [72–74]. In this case, brackish-water was considered as a potential reservoir of *F. tularensis* [78]. Altogether, available data indicate that human contamination with *F. tularensis* may occur from a wide diversity of aquatic sources and activities.

*Francisella* species other than *F. tularensis* are primarily considered aquatic bacteria. *F. philomiragia* and *F. novicida* have been occasionally involved in human infections, especially in immunocompromised patients, but also in specific situations such as near-drowning accident [81–86,89,97–100]. Infections caused by these species were frequently associated with exposure to brackish-water or salt-water [81–86,89,97,99]. In line with this observation, *F. philomiragia* and *F. novicida* have been frequently isolated from brackish- and salt-water samples [78,89,90,92,101,102]. In recent years, novel *Francisella* species have been detected in environmental or clinical samples, the majority of them being associated with an aquatic reservoir. Among them, *F. hispaniensis* [105] and *F. halioticida* [113] are rare human pathogens. These findings strongly suggest that the *Francisella* species are mainly aquatic bacteria.

Several mechanisms could be involved in the survival of *F. tularensis* in aquatic environments. Experimental and epidemiological studies have demonstrated that both *F. tularensis* subsp. *tularensis* and *F. tularensis* subsp. *holarctica* can survive for long periods in water microcosms [32,78,118–120,125,128–132]. Interestingly, after a long stay in water, *F. tularensis* has been shown experimentally to evolve to a VBNC state [118,120], which could account for long-term survival of this bacterium in water. Both type A and type B strains of *F. tularensis* can form biofilms *in vitro* [138,139], another potential survival mechanism of these bacteria in aquatic environments. Experimentally, both subspecies are able to multiply in amoebae or at least interact with these protozoa to enhance their survival [145,147,150,152,153]. Finally, *F. tularensis* subsp. *holarctica* is also able to infect mosquito larvae *in vitro* and survive during larvae maturation up to the adult stage [57,63,138,156,157]*.* It should be mentioned, however, that all these mechanisms have been evaluated experimentally, but not yet confirmed in natural water environments. It is very likely that all these mechanisms exist and are entangled. Infected animals and animal carcasses may contaminate water environments, in which protozoa, mosquito larvae and biofilm communities may become contaminated with *F. tularensis* and serve as reservoirs for this bacterium.

**Figure 1.**
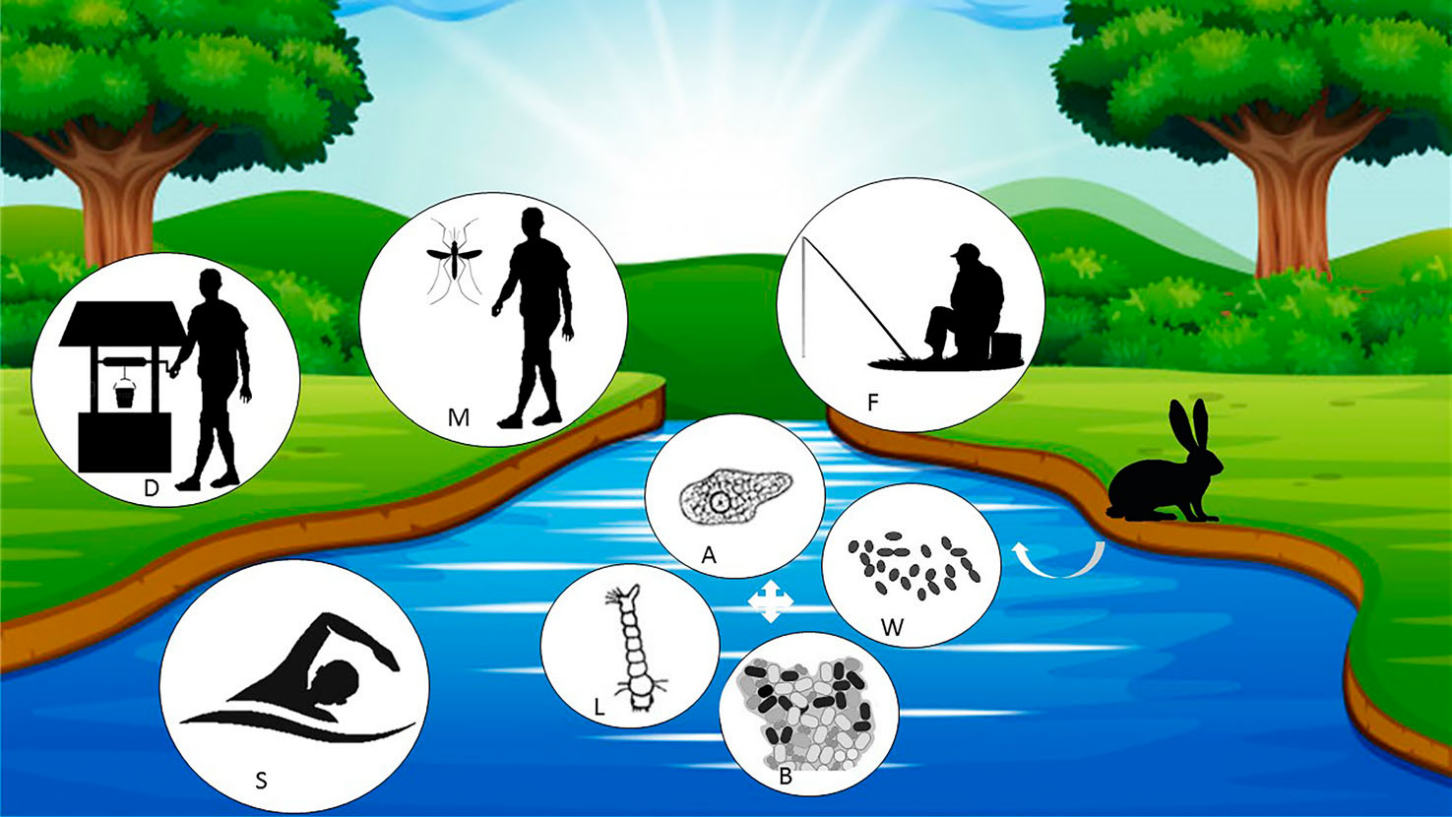
Potential aquatic sources of human infections with *Francisella tularensis*. *Francisella tularensis* is released into water from animals. The bacterium is able to survive in water (W), in mosquito larvae (L), in biofilms (B), or in cooperation with amoeba (A). Human can be contaminated from the aquatic reservoir by drinking contaminated water (D), after a mosquito bite (M), or during swimming (S) and fishing (F) activities.

In conclusion, our goal was to demonstrate that *F. tularensis* is likely able to survive for prolonged periods in various aquatic environments, which likely constitute a significant reservoir for this bacterium. Figure 1 summarizes the probable tularemia aquatic cycle as it can be pictured from current literature data. In terms of public health, it is important to remember that providing people with access to safe drinking water via treatment of municipal and private sources remains a priority. Better characterization and control of the aquatic reservoir of *F. tularensis* would also be of tremendous importance following a bioterrorist attack. Following a bioterrorist attack, *F. tularensis* could survive for months in the environment, leading to a high number of secondary tularemia cases. Water and mosquito reservoirs should be monitored in the overall tularemia surveillance, in addition to the wildlife reservoir.

## References

[1] Sjöstedt A. Tularemia: history, epidemiology, pathogen physiology, and clinical manifestations. Ann N Y Acad Sci. 2007;1105:1–29. 17395726 10.1196/annals.1409.009

[2] Dennis DT, et al. Tularemia as a biological weapon: medical and public health management. JAMA. 2001;285:2763–2773. 11386933 10.1001/jama.285.21.2763

[3] Johansson A, et al. Objections to the transfer of *Francisella novicida* to the subspecies rank of *Francisella tularensis*. Int J Syst Evol Microbiol. 2010;60:1717–1718. 20688748 10.1099/ijs.0.022830-0 PMC7442299

[4] Busse H-J, et al. Objections to the transfer of *Francisella novicida* to the subspecies rank of *Francisella tularensis* - response to Johansson et al. Int J Syst Evol Microbiol. 2010;60:1718–1720. 20688749 10.1099/00207713-60-8-1718

[5] Chaudhuri RR, et al. Genome sequencing shows that European isolates of *Francisella tularensis* subspecies *tularensis* are almost identical to US laboratory strain Schu S4. PloS One. 2007;2:e352. 17406676 10.1371/journal.pone.0000352 PMC1832225

[6] Jackson J, et al. *Francisella tularensis* subspecies *holarctica*, Tasmania, Australia, 2011. Emerg Infect Dis 2012;18:1484–1486. 22931809 10.3201/eid1809.111856 PMC3437722

[7] Gyuranecz M, et al. Investigation of the ecology of *Francisella tularensis* during an inter-epizootic period. Vector Borne Zoonotic Dis Larchmt N. 2011;11:1031–1035. 10.1089/vbz.2010.0091 21142970

[8] Maurin M, Gyuranecz M. Tularaemia: clinical aspects in Europe. Lancet Infect Dis. 2016;16:113–124. 26738841 10.1016/S1473-3099(15)00355-2

[9] Karpoff SP, Antonoff NI. The spread of tularemia through water, as a New Factor in its Epidemiology. J Bacteriol. 1936;32:243–258. 16559947 10.1128/jb.32.3.243-258.1936 PMC543788

[10] Akalin H, Helvaci S, Gedikoğlu S. Re-emergence of tularemia in Turkey. Int J Infect Dis IJID Off Publ Int Soc Infect Dis. 2009;13:547–551. 10.1016/j.ijid.2008.09.020 19119037

[11] Willke A, et al. An outbreak of oropharyngeal tularaemia linked to natural spring water. J Med Microbiol. 2009;58:112–116. 19074661 10.1099/jmm.0.002279-0

[12] Balci E, et al. Tularemia outbreaks in Kayseri, Turkey: an evaluation of the effect of climate change and climate variability on tularemia outbreaks. J Infect Public Health. 2014;7:125–132. 24216516 10.1016/j.jiph.2013.09.002

[13] Meric M, et al. Evaluation of clinical, laboratory, and therapeutic features of 145 tularemia cases: the role of quinolones in oropharyngeal tularemia. APMIS Acta Pathol Microbiol Immunol Scand. 2008;116:66–73. 10.1111/j.1600-0463.2008.00901.x 18254782

[14] Helvaci S, Gedikoğlu S, Akalin H, et al. Tularemia in Bursa, Turkey: 205 cases in ten years. Eur J Epidemiol. 2000;16:271–276. 10870943 10.1023/a:1007610724801

[15] Ulu-Kilic A, Gulen G, Sezen F, et al. Tularemia in central Anatolia. Infection. 2013;41:391–399. 23104256 10.1007/s15010-012-0355-1

[16] Meric M, Sayan M, Dundar D, et al. Tularaemia outbreaks in Sakarya, Turkey: case-control and environmental studies. Singapore Med J. 2010;51:655–659. 20848064

[17] Leblebicioglu H, et al. Outbreak of tularemia: a case-control study and environmental investigation in Turkey. Int J Infect Dis IJID Off Publ Int Soc Infect Dis. 2008;12:265–269. 10.1016/j.ijid.2007.06.013 17983789

[18] Sencan I, et al. An outbreak of oropharyngeal tularemia with cervical adenopathy predominantly in the left side. Yonsei Med J. 2009;50:50–54. 19259348 10.3349/ymj.2009.50.1.50 PMC2649863

[19] Ozdemir D, et al. Comparison of the 2000 and 2005 outbreaks of tularemia in the Duzce region of Turkey. Jpn J Infect Dis. 2007;60:51–52. 17314427

[20] Gürcan S, et al. Tularemia re-emerging in European part of Turkey after 60 years. Jpn J Infect Dis. 2006;59:391–393. 17186960

[21] Gülhan B, Tezer H, Kanık-Yüksek S, et al. A rare cause of abdominal lymphadenopathy–tularemia: report of two pediatric cases. Turk J Pediatr. 2014;56:192–195. 24911857

[22] Yilmaz GR, et al. Tularemia during pregnancy: three cases. Vector Borne Zoonotic Dis Larchmt N. 2014;14:171–173. 10.1089/vbz.2013.1406 24359416

[23] Turhan V, Berber U, Haholu A, et al. Differential diagnosis of cervical lymphadenitis mimicking malignancy due to tularemia: our experiences. Indian J Pathol Microbiol. 2013;56:252–257. 24152503 10.4103/0377-4929.120381

[24] Karagöz S, et al. Francisella tularensis bacteremia: report of two cases and review of the literature. New Microbiol. 2013;36:315–323. 23912874

[25] Yeşilyurt M, Kiliç S, Çelebі B, et al. Tularemia during pregnancy: report of four cases. Scand J Infect Dis. 2013;45:324–328. 22998506 10.3109/00365548.2012.720027

[26] Bilgul M, Mücahit Y, Ertaban DA, et al. A patient with cervical swelling. Intern Med Tokyo Jpn. 2011;50:2253–2254. 10.2169/internalmedicine.50.5715 21963756

[27] Ugur KS, et al. Three cases of oropharyngeal tularemia in Turkey. Auris Nasus Larynx. 2011;38:532–537. 21236608 10.1016/j.anl.2010.12.007

[28] Kandemir B, Erayman I, Bitirgen M, et al. Tularemia presenting with tonsillopharyngitis and cervical lymphadenitis: report of two cases. Scand J Infect Dis. 2007;39:620–622. 17577830 10.1080/00365540601105814

[29] Arikan OK, Koç C, Bozdoğan O. Tularemia presenting as tonsillopharyngitis and cervical lymphadenitis: a case report and review of the literature. Eur Arch Oto-Rhino-Laryngol. 2003;260:298–300. 10.1007/s00405-002-0565-8 12883950

[30] Karadenizli A, Gurcan S, Kolayli F, et al. Outbreak of tularaemia in Golcuk, Turkey in 2005: report of 5 cases and an overview of the literature from Turkey. Scand J Infect Dis 2005;37:712–716. 16191887 10.1080/00365540510012125

[31] Aktas D, et al. Oropharyngeal tularemia outbreak associated with drinking contaminated tap water, Turkey, July-September 2013. Emerg Infect Dis 2015;21:2194–2196. 26584074 10.3201/eid2112.142032 PMC4672448

[32] Karadenizli A, et al. Genomic analyses of Francisella tularensis strains confirm disease transmission from drinking water sources, Turkey, 2008, 2009 and 2012. Euro Surveill Bull Eur Sur Mal Transm Eur Commun Dis Bull 2015;20(21). pii: 21136. 10.2807/1560-7917.es2015.20.21.21136 26062561

[33] Celebi S, Hacimustafaoglu M, Gedikoglu S. Tularemia in children. Indian J Pediatr. 2008;75:1129–1132. 18810348 10.1007/s12098-008-0180-9

[34] Celebi G, et al. Tularemia, a reemerging disease in northwest Turkey: epidemiological investigation and evaluation of treatment responses. Jpn J Infect Dis. 2006;59:229–234. 16936340

[35] Gürcan S, Otkun MT, Otkun M, et al. An outbreak of tularemia in Western Black Sea region of Turkey. Yonsei Med J. 2004;45:17–22. 15004863 10.3349/ymj.2004.45.1.17

[36] Sahin M, Atabay HI, Bicakci Z, et al. Outbreaks of tularemia in Turkey. Kobe J Med Sci. 2007;53:37–42. 17579300

[37] Kilic S, et al. Water as source of *Francisella tularensis* infection in humans, Turkey. Emerg Infect Dis. 2015;21:2213–2216. 26583383 10.3201/eid2112.150634 PMC4672436

[38] Grunow R, Kalaveshi A, Kühn A, et al. Surveillance of tularaemia in Kosovo, 2001 to 2010. Euro Surveill Bull Eur Sur Mal Transm Eur Commun Dis Bull 2012;17(28). pii: 20217. 10.2807/ese.17.28.20217-en 22835441

[39] Reintjes R, et al. Tularemia outbreak investigation in Kosovo: case control and environmental studies. Emerg Infect Dis. 2002;8:69–73. 11749751 10.3201/eid0801.010131 PMC2730257

[40] Kantardjiev T, et al. Tularemia outbreak, Bulgaria, 1997–2005. Emerg Infect Dis. 2006;12:678–680. 16704820 10.3201/eid1204.050709 PMC3294687

[41] Chitadze N, et al. Water-borne outbreak of oropharyngeal and glandular tularemia in Georgia: investigation and follow-up. Infection. 2009;37:514–521. 19826763 10.1007/s15010-009-8193-5

[42] Hristovski KD, Pacemska-Atanasova T, Olson LW, et al. Potential health implications of water resources depletion and sewage discharges in the Republic of Macedonia. J Water Health. 2016;14:682–691. 27441863 10.2166/wh.2016.274

[43] Berdal BP, et al. Field detection of *Francisella tularensis*. Scand J Infect Dis. 2000;32:287–291. 10879600 10.1080/00365540050165938

[44] Brantsaeter AB, Krogh T, Radtke A, et al. Tularaemia outbreak in northern Norway. Euro Surveill Bull Eur Sur Mal Transm Eur Commun Dis Bull. 2007;12:E070329.2. 10.2807/esw.12.13.03165-en 17439796

[45] Larssen KW, et al. Outbreak of tularaemia in central Norway, January to March 2011. Euro Surveill Bull Eur Sur Mal Transm Eur Commun Dis Bull 2011;16(13). pii: 19828. 21489376

[46] Eliasson H, Bäck E. Tularaemia in an emergent area in Sweden: an analysis of 234 cases in five years. Scand J Infect Dis. 2007;39:880–889. 17886125 10.1080/00365540701402970

[47] Lindhusen Lindhé E, Hjertqvist M, Wahab T. Outbreak of tularaemia connected to a contaminated well in the Västra Götaland region in Sweden. Zoonoses Public Health. 2018;65:142–146. 28905501 10.1111/zph.12382

[48] Mignani E, Palmieri F, Fontana M, et al. Italian epidemic of waterborne tularaemia. Lancet Lond Engl. 1988;2:1423. 10.1016/s0140-6736(88)90613-7 2904549

[49] Dlugaiczyk J, et al. Oropharyngeal tularemia–a differential diagnosis of tonsillopharyngitis and cervical lymphadenitis. Wien Klin Wochenschr. 2010;122:110–114. 20213378 10.1007/s00508-009-1274-8

[50] Rydén P, et al. Outbreaks of tularemia in a boreal forest region depends on mosquito prevalence. J Infect Dis. 2012;205:297–304. 22124130 10.1093/infdis/jir732 PMC3244368

[51] Svensson K, et al. Landscape epidemiology of tularemia outbreaks in Sweden. Emerg Infect Dis. 2009;15:1937–1947. 19961673 10.3201/eid1512.090487 PMC3044527

[52] Eliasson H, et al. The 2000 tularemia outbreak: a case-control study of risk factors in disease-endemic and emergent areas, Sweden. Emerg Infect Dis. 2002;8:956–960. 12194773 10.3201/eid0809.020051 PMC2732558

[53] Payne L, Arneborn M, Tegnell A, et al. Endemic tularemia, Sweden, 2003. Emerg Infect Dis 2005;11:1440–1442. 16229776 10.3201/eid1109.041189 PMC3310613

[54] Wik O. Large tularaemia outbreak in Varmland, central Sweden, 2006. Euro Surveill Bull Eur Sur Mal Transm Eur Commun Dis Bull 2006;11:E060921.1. 10.2807/esw.11.38.03052-en 17075151

[55] Desvars A, et al. Epidemiology and ecology of tularemia in Sweden, 1984–2012. Emerg Infect Dis 2015;21:32–39. 25529978 10.3201/eid2101.140916 PMC4285262

[56] Desvars-Larrive A, et al. High-risk regions and outbreak modelling of tularemia in humans. Epidemiol Infect. 2017;145:482–490. 27806741 10.1017/S0950268816002478 PMC9507643

[57] Thelaus J, et al. Francisella tularensis subspecies holarctica occurs in Swedish mosquitoes, persists through the developmental stages of laboratory-infected mosquitoes and is transmissible during blood feeding. Microb Ecol. 2014;67:96–107. 24057273 10.1007/s00248-013-0285-1 PMC3907667

[58] Jounio U, Renko M, Uhari M. An outbreak of holarctica-type tularemia in pediatric patients. Pediatr Infect Dis J. 2010;29:160–162. 19918211 10.1097/INF.0b013e3181b9a6b4

[59] Karhukorpi EK, Karhukorpi J. Rapid laboratory diagnosis of ulceroglandular tularemia with polymerase chain reaction. Scand J Infect Dis. 2001;33:383–385. 11440227 10.1080/003655401750174101

[60] Ylipalosaari P, Ala-Kokko TI, Tuominen H, et al. Guillain-Barré syndrome and ulceroglandular tularemia. Infection. 2013;41:881–883. 23712689 10.1007/s15010-013-0466-3

[61] Rossow H, et al. Incidence and seroprevalence of tularaemia in Finland, 1995 to 2013: regional epidemics with cyclic pattern. Euro Surveill Bull Eur Sur Mal Transm Eur Commun Dis Bull 2015;20:21209. 10.2807/1560-7917.es2015.20.33.21209 26314404

[62] Hanke CA, et al. Ulceroglandular tularemia in a toddler in Germany after a mosquito bite. Eur J Pediatr. 2009;168:937–940. 19132387 10.1007/s00431-008-0862-3

[63] Triebenbach AN, et al. Detection of *Francisella tularensis* in Alaskan mosquitoes (Diptera: Culicidae) and assessment of a laboratory model for transmission. J Med Entomol. 2010;47:639–648. 20695280 10.1603/me09192 PMC3590900

[64] Ughetto E, et al. An original case of *Francisella tularensis* subsp. *holarctica* bacteremia after a near-drowning accident. Infect Dis Lond Engl. 2015;47:588–590. 10.3109/23744235.2015.1028099 25816922

[65] Väyrynen SA, et al. Pneumonic tularaemia: experience of 58 cases from 2000 to 2012 in Northern Finland. Infect Dis Lond Engl. 2017;49:758–764. 10.1080/23744235.2017.1341054 28618894

[66] Bayhan-Taş GI, Tanir G, Celebi B. Two cases of glandular tularemia from Turkey. Turk J Pediatr. 2012;54:203–206. 22734313

[67] Peker E, Ayaydin A, Duran N. Familial tularaemia. Indian J Med Microbiol 2009;27:272–275. 19584516 10.4103/0255-0857.53217

[68] Guerpillon B, et al. Keep an Ear Out for *Francisella tularensis*: Otomastoiditis cases after Canyoneering. Front Med. 2016;3:9. 10.3389/fmed.2016.00009 PMC4776157 26973838

[69] Anda P, et al. Waterborne outbreak of tularemia associated with crayfish fishing. Emerg Infect Dis. 2001;7:575–582. 11485678 10.3201/eid0707.010740 PMC2631832

[70] Jassal DS, Targownik L, Thottingal P, et al. Photo quiz. Diagnosis: tularemia. Clin Infect Dis 1999;29(275):420–421. 10.1086/520197 10476724

[71] Whitten T, et al. Notes from the field: *Francisella tularensis* type B infection from a fish Hook injury - Minnesota, 2016. MMWR Morb Mortal Wkly Rep 2017;66:194. 28231234 10.15585/mmwr.mm6607a3 PMC5657846

[72] Teutsch SM, et al. Pneumonic tularemia on Martha’s Vineyard. N Engl J Med. 1979;301:826–828. 481515 10.1056/NEJM197910113011507

[73] Feldman KA, et al. An outbreak of primary pneumonic tularemia on Martha’s Vineyard. N Engl J Med. 2001;345:1601–1606. 11757506 10.1056/NEJMoa011374

[74] Matyas BT, Nieder HS, Telford SR. Pneumonic tularemia on Martha’s Vineyard: clinical, epidemiologic, and ecological characteristics. Ann N Y Acad Sci. 2007;1105:351–377. 17442781 10.1196/annals.1409.013

[75] Berrada ZL, Goethert HK, Telford SR. Raccoons and skunks as sentinels for enzootic tularemia. Emerg Infect Dis. 2006;12:1019–1021. 16707067 10.3201/eid1206.05879 PMC3373054

[76] Feldman KA, et al. Tularemia on Martha’s Vineyard: seroprevalence and occupational risk. Emerg Infect Dis. 2003;9:350–354. 12643831 10.3201/eid0903.020462 PMC2958548

[77] Goethert HK, Shani I, Telford SR. Genotypic diversity of *Francisella tularensis* infecting *Dermacentor variabilis* ticks on Martha’s Vineyard, Massachusetts. J Clin Microbiol. 2004;42:4968–4973. 15528681 10.1128/JCM.42.11.4968-4973.2004 PMC525218

[78] Berrada ZL, Telford SR. Diversity of *Francisella* species in environmental samples from Martha’s Vineyard, Massachusetts. Microb Ecol. 2010;59:277–283. 19669828 10.1007/s00248-009-9568-y PMC2836248

[79] Messier V, et al. Seroprevalence of seven zoonotic infections in Nunavik, Quebec (Canada). Zoonoses Public Health. 2012;59:107–117. 21824376 10.1111/j.1863-2378.2011.01424.x

[80] Sjödin A, et al. Genome characterisation of the genus *Francisella* reveals insight into similar evolutionary paths in pathogens of mammals and fish. BMC Genomics. 2012;13:268. 22727144 10.1186/1471-2164-13-268 PMC3485624

[81] Friis-Møller A, Lemming LE, Valerius NH, et al. Problems in identification of *Francisella philomiragia* associated with fatal bacteremia in a patient with chronic granulomatous disease. J Clin Microbiol. 2004;42:1840–1842. 15071065 10.1128/JCM.42.4.1840-1842.2004 PMC387557

[82] Polack FP, Harrington SM, Winkelstein JA, et al. Recurrent *Francisella philomiragia* sepsis in chronic granulomatous disease. Pediatr Infect Dis J. 1998;17:442–443. 10.1097/00006454-199805000-00028 9613671

[83] Robles-Marhuenda A, et al. *Francisella philomiragia*: Think of chronic granulomatous disease. J Clin Immunol. 2018;38:257–259. 29663155 10.1007/s10875-018-0498-7

[84] Mailman TL, Schmidt MH. *Francisella philomiragia* adenitis and pulmonary nodules in a child with chronic granulomatous disease. Can J Infect Dis Med Microbiol J Can Mal Infect Microbiol Medicale. 2005;16:245–248. 10.1155/2005/486417 PMC2095034 18159552

[85] Sicherer SH, Asturias EJ, Winkelstein JA, et al. *Francisella philomiragia* sepsis in chronic granulomatous disease. Pediatr Infect Dis J. 1997;16:420–422. 9109152 10.1097/00006454-199704000-00021

[86] Wenger JD, et al. Infection caused by *Francisella philomiragia* (formerly *Yersinia philomiragia*). A newly recognized human pathogen. Ann Intern Med. 1989;110:888–892. 2541646 10.7326/0003-4819-110-11-888

[87] Kreitmann L, et al. Disseminated infection caused by *Francisella philomiragia*, France, 2014. Emerg Infect Dis 2015;21:2260–2261. 26583375 10.3201/eid2112.150615 PMC4672438

[88] Relich RF, et al. *Francisella philomiragia* bacteremia in a patient with acute respiratory insufficiency and acute-on-chronic kidney disease. J Clin Microbiol. 2015;53:3947–3950. 26400786 10.1128/JCM.01762-15 PMC4652090

[89] Whitehouse CA, Kesterson KE, Duncan DD, et al. Identification and characterization of *Francisella* species from natural warm springs in Utah, USA. Lett Appl Microbiol. 2012;54:313–324. 22283482 10.1111/j.1472-765X.2012.03214.x

[90] Brinkmeyer R. Diversity of bacteria in ships ballast water as revealed by next generation DNA sequencing. Mar Pollut Bull. 2016;107:277–285. 27076378 10.1016/j.marpolbul.2016.03.058

[91] Gu Q, et al. Characterization of *Francisella* species isolated from the cooling water of an air conditioning system. Braz J Microbiol Publ Braz Soc Microbiol. 2015;46:921–927. 10.1590/S1517-838246320140465 PMC4568874 26413079

[92] Duodu S, Larsson P, Sjödin A, et al. The distribution of *Francisella*-like bacteria associated with coastal waters in Norway. Microb Ecol. 2012;64:370–377. 22370877 10.1007/s00248-012-0023-0

[93] Barns SM, Grow CC, Okinaka RT, et al. Detection of diverse new *Francisella*-like bacteria in environmental samples. Appl Environ Microbiol. 2005;71:5494–5500. 16151142 10.1128/AEM.71.9.5494-5500.2005 PMC1214603

[94] Jensen WI, Owen CR, Jellison WL. *Yersinia philomiragia* sp. nov., a new member of the *Pasteurella* group of bacteria, naturally pathogenic for the muskrat (Ondatra zibethica). J Bacteriol. 1969;100:1237–1241. 5361214 10.1128/jb.100.3.1237-1241.1969 PMC250302

[95] Bonnet S, et al. Prevalence of tick-borne pathogens in adult *Dermacentor* spp. ticks from nine collection sites in France. Vector Borne Zoonotic Dis Larchmt N. 2013;13:226–236. 10.1089/vbz.2011.0933 23421886

[96] Kingry LC, Petersen JM. Comparative review of *Francisella tularensis* and *Francisella novicida*. Front Cell Infect Microbiol. 2014;4:35. 24660164 10.3389/fcimb.2014.00035 PMC3952080

[97] Brett M, et al. *Francisella novicida* bacteremia after a near-drowning accident. J Clin Microbiol. 2012;50:2826–2829. 22692740 10.1128/JCM.00995-12 PMC3421515

[98] Brett ME, et al. Outbreak of *Francisella novicida* bacteremia among inmates at a louisiana correctional facility. Clin Infect Dis Off Publ Infect Dis Soc Am. 2014;59:826–833. 10.1093/cid/ciu430 24944231

[99] Whipp MJ, et al. Characterization of a *novicida*-like subspecies of *Francisella tularensis* isolated in Australia. J Med Microbiol. 2003;52:839–842. 12909664 10.1099/jmm.0.05245-0

[100] Clarridge JE, et al. Characterization of two unusual clinically significant *Francisella* strains. J Clin Microbiol. 1996;34:1995–2000. 8818897 10.1128/jcm.34.8.1995-2000.1996 PMC229169

[101] Petersen JM, et al. Direct isolation of *Francisella* spp. from environmental samples. Lett Appl Microbiol. 2009;48:663–667. 19413814 10.1111/j.1472-765X.2009.02589.x

[102] Challacombe JF, et al. Whole-genome relationships among *Francisella* bacteria of diverse origins define new species and provide specific regions for detection. Appl Environ Microbiol. 2017;83:e02589–16. 27881415 10.1128/AEM.02589-16 PMC5244304

[103] Escudero R, et al. A possible novel *Francisella* genomic species isolated from blood and urine of a patient with severe illness. Clin Microbiol Infect Off Publ Eur Soc Clin Microbiol Infect Dis. 2010;16:1026–1030. 10.1111/j.1469-0691.2009.03029.x 19709068

[104] Huber B, et al. Description of *Francisella hispaniensis* sp. nov., isolated from human blood, reclassification of *Francisella novicida* (Larson et al. 1955) Olsufiev et al. 1959 as *Francisella tularensis* subsp. *novicida* comb. nov. and emended description of the genus *Francisella*. Int J Syst Evol Microbiol. 2010;60:1887–1896. 19783615 10.1099/ijs.0.015941-0

[105] Aravena-Román M, Merritt A, Inglis TJJ. First case of *Francisella* bacteraemia in Western Australia. New Microbes New Infect. 2015;8:75–77. 26649180 10.1016/j.nmni.2015.10.004 PMC4644258

[106] Colquhoun DJ, Duodu S. *Francisella* infections in farmed and wild aquatic organisms. Vet Res. 2011;42:47. 21385413 10.1186/1297-9716-42-47 PMC3060124

[107] Ottem KF, Nylund A, Karlsbakk E, et al. Elevation of *Francisella philomiragia* subsp. *noatunensis* Mikalsen et al. (2007) to *Francisella noatunensis* comb. nov. [syn. *Francisella piscicida* Ottem et al. (2008) syn. nov]. and characterization of *Francisella noatunensis* subsp. *orientalis* subsp. nov., two important fish pathogens. J Appl Microbiol 2009;106:1231–1243. 19187160 10.1111/j.1365-2672.2008.04092.x

[108] Mikalsen J, Olsen AB, Tengs T, et al. *Francisella philomiragia* subsp. *noatunensis* subsp. nov., isolated from farmed Atlantic cod (*Gadus morhua* L.). Int J Syst Evol Microbiol. 2007;57:1960–1965. 17766855 10.1099/ijs.0.64765-0

[109] Ottem KF, et al. New species in the genus *Francisella* (Gammaproteobacteria; *Francisellaceae*); *Francisella piscicida* sp. nov. isolated from cod (*Gadus morhua*). Arch Microbiol. 2007;188:547–550. 17619856 10.1007/s00203-007-0274-1

[110] Brevik OJ, Ottem KF, Kamaishi T, et al. *Francisella halioticida* sp. nov., a pathogen of farmed giant abalone (*Haliotis gigantea*) in Japan. J Appl Microbiol. 2011;111:1044–1056. 21883728 10.1111/j.1365-2672.2011.05133.x

[111] Meyer GR, Lowe GJ, Gilmore SR, et al. Disease and mortality among Yesso scallops *Patinopecten yessoensis* putatively caused by infection with *Francisella halioticida*. Dis Aquat Organ. 2017;125:79–84. 28627495 10.3354/dao03130

[112] Soto E, et al. *Francisella marina* sp. nov., Etiologic agent of Systemic disease in cultured spotted rose snapper (*Lutjanus guttatus*) in Central America. Appl Environ Microbiol. 2018;84(16). pii: e00144–18. 29915103 10.1128/AEM.00144-18 PMC6070750

[113] Respicio-Kingry LB, et al. Cutaneous infection caused by a novel *Francisella* sp. J Clin Microbiol. 2013;51:3456–3460. 23903547 10.1128/JCM.01105-13 PMC3811661

[114] Sjödin A, et al. Complete genome sequence of *Francisella endociliophora* strain FSC1006, isolated from a laboratory culture of the marine ciliate *Euplotes raikovi*. Genome Announc. 2014;2(6):e01227–14. 25428973 10.1128/genomeA.01227-14 PMC4246165

[115] Qu P-H, et al. *Allofrancisella inopinata* gen. nov., sp. nov. and *Allofrancisella frigidaquae* sp. nov., isolated from water-cooling systems, and transfer of *Francisella guangzhouensis* Qu et al. 2013 to the new genus as *Allofrancisella guangzhouensis* comb. nov. Int J Syst Evol Microbiol 2016;66:4832–4838. 27543089 10.1099/ijsem.0.001437

[116] Rydzewski K, et al. Genome sequence and phenotypic analysis of a first German *Francisella* sp. isolate (W12–1067) not belonging to the species *Francisella tularensis*. BMC Microbiol. 2014;14:169. 24961323 10.1186/1471-2180-14-169 PMC4230796

[117] Qu P-H, et al. *Francisella guangzhouensis* sp. nov., isolated from air-conditioning systems. Int J Syst Evol Microbiol. 2013;63:3628–3635. 23606480 10.1099/ijs.0.049916-0

[118] Gilbert SE, Rose LJ. Survival and persistence of nonspore-forming biothreat agents in water. Lett Appl Microbiol. 2012;55:189–194. 22725260 10.1111/j.1472-765X.2012.03277.x

[119] Berrada ZL, Telford Iii SR. Survival of *Francisella tularensis* type A in brackish-water. Arch Microbiol. 2011;193:223–226. 21136042 10.1007/s00203-010-0655-8 PMC3962107

[120] Forsman M, Henningson EW, Larsson E, et al. *Francisella tularensis* does not manifest virulence in viable but non-culturable state. FEMS Microbiol Ecol. 2000;31:217–224. 10719202 10.1111/j.1574-6941.2000.tb00686.x

[121] Oliver JD. The viable but non-culturable state in the human pathogen *Vibrio vulnificus*. FEMS Microbiol Lett. 1995;133:203–208. 8522135 10.1111/j.1574-6968.1995.tb07885.x

[122] Xu HS, et al. Survival and viability of nonculturable *Escherichia coli* and *Vibrio cholerae* in the estuarine and marine environment. Microb Ecol. 1982;8:313–323. 24226049 10.1007/BF02010671

[123] Rollins DM, Colwell RR. Viable but nonculturable stage of *Campylobacter jejuni* and its role in survival in the natural aquatic environment. Appl Environ Microbiol. 1986;52:531–538. 3767358 10.1128/aem.52.3.531-538.1986 PMC203568

[124] Steinert M, Emödy L, Amann R, et al. Resuscitation of viable but nonculturable *Legionella pneumophila* Philadelphia JR32 by *Acanthamoeba castellanii*. Appl Environ Microbiol. 1997;63:2047–2053. 9143134 10.1128/aem.63.5.2047-2053.1997 PMC168494

[125] Williamson DR, et al. A Single mechanosensitive channel protects *Francisella tularensis* subsp. *holarctica* from hypoosmotic shock and promotes survival in the aquatic environment. Appl Environ Microbiol. 2018;84(5). pii: e02203–17. 29269496 10.1128/AEM.02203-17 PMC5812925

[126] Soto E, Revan F. Culturability and persistence of *Francisella noatunensis* subsp. *orientalis* (syn. *Francisella asiatica*) in sea- and freshwater microcosms. Microb Ecol. 2012;63:398–404. 21881943 10.1007/s00248-011-9932-6

[127] Duodu S, Colquhoun D. Monitoring the survival of fish-pathogenic *Francisella* in water microcosms. FEMS Microbiol Ecol. 2010;74:534–541. 20977492 10.1111/j.1574-6941.2010.00973.x

[128] Janse I, van der Plaats RQJ, de Roda Husman AM, et al. Environmental surveillance of zoonotic *Francisella tularensis* in the Netherlands. Front Cell Infect Microbiol. 2018;8:140. 29868496 10.3389/fcimb.2018.00140 PMC5951967

[129] Hightower J, et al. Historical distribution and host-vector diversity of *Francisella tularensis*, the causative agent of tularemia, in Ukraine. Parasit Vectors. 2014;7:453–459. 25318562 10.1186/s13071-014-0453-2 PMC4200231

[130] Simşek H, Taner M, Karadenizli A, et al. Identification of *Francisella tularensis* by both culture and real-time TaqMan PCR methods from environmental water specimens in outbreak areas where tularemia cases were not previously reported. Eur J Clin Microbiol Infect Dis. 2012;31:2353–2357. 22382819 10.1007/s10096-012-1576-z

[131] Broman T, et al. Molecular detection of persistent *Francisella tularensis* subspecies *holarctica* in natural waters. Int J Microbiol. 2011;2011. pii: 851946. 20885922 10.1155/2011/851946 PMC2946586

[132] Kaysser P, et al. Re-emergence of tularemia in Germany: presence of *Francisella tularensis* in different rodent species in endemic areas. BMC Infect Dis. 2008;8:157. 19014635 10.1186/1471-2334-8-157 PMC2629769

[133] van Hoek ML. Biofilms: an advancement in our understanding of *Francisella* species. Virulence. 2013;4:833–846. 24225421 10.4161/viru.27023 PMC3925715

[134] Huq A, Whitehouse CA, Grim CJ, et al. Biofilms in water, its role and impact in human disease transmission. Curr Opin Biotechnol. 2008;19:244–247. 18524568 10.1016/j.copbio.2008.04.005

[135] Lau HY, Ashbolt NJ. The role of biofilms and protozoa in *Legionella pathogenesis*: implications for drinking water. J Appl Microbiol. 2009;107:368–378. 19302312 10.1111/j.1365-2672.2009.04208.x

[136] Percival SL, Thomas JG. Transmission of *Helicobacter pylori* and the role of water and biofilms. J Water Health. 2009;7:469–477. 19491497 10.2166/wh.2009.070

[137] Costerton JW. Cystic fibrosis pathogenesis and the role of biofilms in persistent infection. Trends Microbiol. 2001;9:50–52. 11173226 10.1016/s0966-842x(00)01918-1

[138] Mahajan UV, Gravgaard J, Turnbull M, et al. Larval exposure to *Francisella tularensis* LVS affects fitness of the mosquito *Culex quinquefasciatus*. FEMS Microbiol Ecol. 2011;78:520–530. 22066999 10.1111/j.1574-6941.2011.01182.x

[139] Margolis JJ, et al. Contributions of *Francisella tularensis* subsp. *novicida* chitinases and Sec secretion system to biofilm formation on chitin. Appl Environ Microbiol. 2010;76:596–608. 19948864 10.1128/AEM.02037-09 PMC2805214

[140] Durham-Colleran MW, Verhoeven AB, van Hoek ML. *Francisella novicida* forms in vitro biofilms mediated by an orphan response regulator. Microb Ecol. 2010;59:457–465. 19763680 10.1007/s00248-009-9586-9

[141] Dean RE, Ireland PM, Jordan JE, et al. Rela regulates virulence and intracellular survival of *Francisella novicida*. Microbiol Read Engl. 2009;155:4104–4113. 10.1099/mic.0.031021-0 19762448

[142] Verhoeven AB, Durham-Colleran MW, Pierson T, et al. *Francisella philomiragia* biofilm formation and interaction with the aquatic protist *Acanthamoeba castellanii*. Biol Bull. 2010;219:178–188. 20972262 10.1086/BBLv219n2p178

[143] Soto E, Halliday-Simmonds I, Francis S, et al. Biofilm formation of *Francisella noatunensis* subsp. *orientalis*. Vet Microbiol. 2015;181:313–317. 26507830 10.1016/j.vetmic.2015.10.007

[144] Greub G, Raoult D. Microorganisms resistant to free-living amoebae. Clin Microbiol Rev. 2004;17:413–433. 15084508 10.1128/CMR.17.2.413-433.2004 PMC387402

[145] Buse HY, Schaefer Iii FW, Rice EW. Enhanced survival but not amplification of *Francisella* spp. in the presence of free-living amoebae. Acta Microbiol Immunol Hung. 2017;64:17–36. 27929353 10.1556/030.63.2016.015 PMC7357732

[146] Santic M, et al. Intra-Vacuolar Proliferation of *F. novicida* within *H. vermiformis*. Front Microbiol. 2011;2:78. 21747796 10.3389/fmicb.2011.00078 PMC3128938

[147] Thelaus J, et al. Influence of nutrient status and grazing pressure on the fate of *Francisella tularensis* in lake water. FEMS Microbiol Ecol. 2009;67:69–80. 19120459 10.1111/j.1574-6941.2008.00612.x

[148] Ozanic M, et al. *F. novicida*-infected *A. castellanii* does Not enhance bacterial virulence in mice. Front Cell Infect Microbiol. 2016;6:56. 27242974 10.3389/fcimb.2016.00056 PMC4870235

[149] Lampe EO, et al. Dissection of *Francisella*-host Cell interactions in *Dictyostelium discoideum*. Appl Environ Microbiol. 2015;82:1586–1598. 26712555 10.1128/AEM.02950-15 PMC4771330

[150] El-Etr SH, et al. *Francisella tularensis* type A strains cause the rapid encystment of *Acanthamoeba castellanii* and survive in amoebal cysts for three weeks postinfection. Appl Environ Microbiol. 2009;75:7488–7500. 19820161 10.1128/AEM.01829-09 PMC2786426

[151] Lauriano CM, et al. Mgla regulates transcription of virulence factors necessary for *Francisella tularensis* intraamoebae and intramacrophage survival. Proc Natl Acad Sci U S A. 2004;101:4246–4249. 15010524 10.1073/pnas.0307690101 PMC384726

[152] Berdal BP, Mehl R, Meidell NK, et al. Field investigations of tularemia in Norway. FEMS Immunol Med Microbiol. 1996;13:191–195. 8861027 10.1111/j.1574-695X.1996.tb00235.x

[153] Abd H, Johansson T, Golovliov I, et al. Survival and growth of *Francisella tularensis* in *Acanthamoeba castellanii*. Appl Environ Microbiol. 2003;69:600–606. 12514047 10.1128/AEM.69.1.600-606.2003 PMC152416

[154] Gustafsson K. Growth and survival of four strains of *Francisella tularensis* in a rich medium preconditioned with *Acanthamoeba palestinensis*. Can J Microbiol. 1989;35:1100–1104. 2630032 10.1139/m89-184

[155] Thomas V, Herrera-Rimann K, Blanc DS, et al. Biodiversity of amoebae and amoeba-resisting bacteria in a hospital water network. Appl Environ Microbiol. 2006;72:2428–2438. 16597941 10.1128/AEM.72.4.2428-2438.2006 PMC1449017

[156] Bäckman S, Näslund J, Forsman M, et al. Transmission of tularemia from a water source by transstadial maintenance in a mosquito vector. Sci Rep. 2015;5:7793. 25609657 10.1038/srep07793 PMC4302321

[157] Lundström JO, et al. Transstadial transmission of *Francisella tularensis holarctica* in mosquitoes, Sweden. Emerg Infect Dis. 2011;17:794–799. 21529386 10.3201/eid1705.100426 PMC3321753

[158] Andersen LK, Davis MDP. Climate change and the epidemiology of selected tick-borne and mosquito-borne diseases: update from the International Society of Dermatology Climate Change Task Force. Int J Dermatol. 2017;56:252–259. 27696381 10.1111/ijd.13438

